# Bibliographical Notices

**Published:** 1844-04-01

**Authors:** 


### Periscope;

OR,

### CIRCUMSPECTIVE REVIEW.

" Ore trahit quodcunque potest, atque addit acervo."

### Notices of some New Works.

### Observations and Reflections on some rare Varieties of Dislocations. By *F. Bouisson.*

M. Bouisson, Pathological Professor at Montpellier, has published a paper with this heading in the Annales de la Chirurgie. The observations appear to be principally suggested by some interesting specimens of pathological anatomy which have come under his notice.

1. *Dislocation of the 4th Thoracic Cartilage at its Costal Articulation.*—A man named Boudet was thrown from an ass in the month of January, 1838. Whilst Boudet was lying on his back, the animal trod upon his breast, the pressure being exerted principally upon the costal articulation of the 4th cartilage, which was driven in towards the cavity of the chest. When seen by the surgeon, the man experienced some pain and oppression about the seat of the injury. There was no contusion or ecchymosis, and it was easy to determine the exact nature of the lesion. The 4th costal cartilage was depressed backwards and downwards; the anterior extremity of the corresponding rib caused a slight projection externally, which, however, presented no inequality which could give rise to the idea of a fracture near the joint. On the patient's making a deep inspiration, the cartilage became replaced of itself, but was again luxated during expiration. The treatment consisted merely in applying a bandage sufficiently tight to render the parietes of the chest immoveable. The man recovered perfectly, and has since experienced no inconvenience from the accident.

M. Bouisson remarks that luxations of the costal cartilages appear to be but little known. Sir A. Cooper, indeed, states that sometimes a cartilage is separated from the anterior extremity of the rib, but adds that the cartilage then projects beyond the rib. In the present instance the projection, it will be seen, occurred in the opposite direction, and such, M. Bouisson thinks, must always be the case, especially if the seat of the luxation be in one of the five or six upper ribs. The triangularis sterni is inserted by digitations into the external extremities of the upper costal cartilages, and its contraction during expiration must draw them downwards and inwards with the more force as this muscle acts upon the extremity of the lever represented by the luxated cartilage. In inspiration, on the contrary, the triangularis sterni is relaxed, and the elevation of the cartilage is owing to the contraction of the pectoralis major which is inserted into its external surface. But this muscle acts under very unfavourable circumstances as regards the cartilages, and consequently a forced inspiration is requisite to enable one of these, if luxated, to return to its natural situation.

2. *Imperfect Dislocation upwards and outwards of the Acromial Extremity of the Clavicle.*—M. D——, in the month of July, 1842, fell in getting down from a diligence, striking the top of his right shoulder. Acute pain was immediately experienced, together with a sensation as if something had given way. On examining the part, the external extremity of the clavicle was found to project slightly above the acromion, and to be dislocated upwards and outwards; but the displacement was very limited, the tip of the shoulder presenting no very apparent deformity. The patient could raise the arm and move it in every direction; but this exercise occasioned acute pain, and increased or diminished the amount of the deformity according to the direction of the motion. It was very easy to remove the displacement, by acting on the humerus, and carrying the tip of the shoulder upwards and outwards, and then making slight pressure on the extremity of the clavicle—this soon regained its normal position; the reduction was maintained by Dessault's apparatus. The patient did well.

M. Bouisson is of opinion that, in this case, the acromio-clavicular ligaments were ruptured, but that the force had not been sufficiently great to tear the coraco-clavicular ligaments, the thickness and resistance of which maintained the clavicle nearly in its original position. In ordinary cases of dislocation of the clavicle upwards and outwards, the displacement is much more marked, because *all* the ligaments which unite the clavicle to the scapula are lacerated; the clavicle is then easily carried upwards by the action of the trapezius, whilst the scapula is drawn towards the trunk, either by the effect of the blow itself, or by muscular contraction. Hence it follows that luxations of the clavicle in this direction may be said to be *complete*, when both sets of ligaments are ruptured; *incomplete* or *imperfect*, when the coraco-clavicular ligaments remain perfect.

3. *Sub-acromial Luxation of the Left Humerus, the Articular Surface of this Bone being directed outwards.*—In the collection of M. Dubrueil exists a preparation, of which the following is a description.

The humerus is turned round, and appears to have described a half-rotation upon its axis, so that its head or articular surface looks directly outwards and upwards, whilst the external tuberosity looks backwards and inwards. The bone is neither elevated nor depressed, but has been transported directly outwards during rotation, and has not passed the level of the posterior border of the acromion. The bicipital groove is placed behind, and corresponds to the external border of the glenoid fossa. The great tuberosity rests upon the external half of this same cavity, and presents traces of a superficial separation of osseous substance. The detached fragment is placed between the glenoid cavity and the head of the humerus. The capsular ligament is torn to a considerable extent at its humeral insertion, so that the articular head of the humerus can be seen external to the capsule. There is no trace of any false joint; the glenoid cavity and head of the humerus present their usual form and dimensions.

Unfortunately there is no history belonging to the preparation; but, as far as we can judge from the facts furnished by morbid anatomy, we may form the following ideas with regard to the case.

The luxation was not the effect of a fall or a blow, but it is probable that the arm, being seized by a machine, had undergone a rotation on its axis from within outwards. In this movement, the capsule being stretched on the cartilaginous demi-sphere of the humerus, was torn at the level of the anatomical neck, and the gyration of the bone continuing, so as to force the external tuberosity on the glenoid cavity, the muscles which are inserted into this process contributed to the separation of the portion of bone.

This luxation must have produced symptoms differing from those of the ordinary luxations. Here the top of the shoulder would preserve its habitual roundness; the arm placed by the side of, and parallel to, the trunk, would retain its ordinary length; but the palm of the hand would look outwards, the olecranon would be turned towards the corresponding side of the trunk; the transverse diameter of the humero-cubital articulation would be directed from before backwards. With regard to the means of reduction, it would probably be necessary to act upon the limb in the opposite direction to the luxating cause. The scapula must be fixed, and the arm, seized by both hands, without making any traction upon it, must be rotated from without inwards, so as to bring the parts into their proper relations.

This dislocation is called by M. Bouisson the "luxation par renversement."

4. *Incomplete Luxation of the Upper Extremity of the Ulna forwards.*—A preparation in the possession of M. Dubrueil presents the following appearances.

The humerus of the right side is the seat of an oblique fracture directed downwards and inwards from the level of the epicondyle to the trochlea of the articular surface; there is, consequently, an internal portion continuous with the humerus, and directed very obliquely; and an external portion completely detached from the bone, and comprising the surface for the radius and the middle pulley of the articulation. This fragment is articulated with the radius by its lower border, is in contact with the great sygmoid cavity by its internal angle, and is connected by pseudarthrosis with the portion of the humerus which corresponds to the fracture. It is thus separated from the trochlea of the humerus by an interval in which is lodged the olecranon process. All the articular surface of the ulna is free, and measures by its transverse extent the amount of separation of the trochlea and the external fragment. The posterior fossa of the humerus is effaced, and this bone is united, by its fractured surface, to the upper extremity of the olecranon. Hence the olecranon, instead of being situated behind the humerus, is on the same plane with it in the interval produced by the fracture; and consequently the deposition of the parts represents an oblique fracture of the humerus with incomplete luxation of the ulna forwards.

5. *Simultaneous Luxation of the Bones of the Fore-arm backwards, and of the Lower End of the Ulna in the same direction. Fracture of the Body of the Radius.*—In the Winter of 1838, there was brought to the dissecting-room at Montpellier, the body of a man who had several years previously experienced an injury of the arm from a fall from a considerable height. No attempt had been made to reduce the parts at the time. On examination after death, both bones of the fore-arm were found to be dislocated on the humerus; the olecranon being very prominent posteriorly, and separated from the humerus by the whole thickness of the coronoid process. The radius is drawn up with the ulna, with which it retains its natural relations. The radius was fractured at its middle part. The lower extremity of the ulna was also dislocated backwards. The small internal articular surface of the radius was free and corresponded by its posterior border only to a small part of the circumference of the head of the displaced bone. The triangular fibro-cartilage was only incompletely torn, and still adhered by a few fibres to the radius.

6. *Luxation of the Femur directly backwards: Double Pseudarthrosis of the Head of this Bone with the lowest part of the Iliac Fossa, and of the Small Trochanter with the Outer Side of the Cotyloid Cavity.*—This preparation is in the museum at Montpelier. The femur on the right side fixed to the innominatum in the direction indicated by the two false articulations, is bent on the pelvis at a right angle; its lower extremity is directed towards the median line, *and the whole bone is rotated outwards,* so that the great trochanter is carried backwards and outwards. The head of the femur, slightly deformed, is sustained by a very short neck, which corresponds to the upper part of the osseous surface which separates the sciatic notch from the cotyloid cavity. About this point the head is received in a circular depression of the ilium, which presents some rugosities on its surface, and is limited by an osseous ridge thicker in front than behind. Some remains of fibrous bands can be distinguished, to which it gave insertion, and which served to fix the head of the femur in its anormal situation. The second false joint exists between the lesser trochanter and the external side of the cotyloid cavity, the trochanter having been brought into this situation by consequence of the projection of the head of the femur and the rotation of the bone outwards. The cotyloid cavity is partly obliterated, and is now triangular in shape.

M. Bouisson remarks, that we may now distinguish three dislocations of the head of the femur backwards. If a line be drawn from the anterior inferior spine of the ilium to the corresponding posterior iliac spine, then if the head of the femur is situated above this line, there is luxation upwards and backwards, or on the dorsum ilii: this is the most common of all the dislocations of the head of the femur. If the head of the bone be thrown below this line, then the luxation may be either backwards and a little downwards, the *ischiatic* variety of M. Gerdy, in which the head of the femur, the capsule having been ruptured, is depressed to the level of the tuberosity of the ischium: or it may be directly backwards either into the ischiatic notch; or, as in this example, on the convex surface which separates the notch from the cotyloid cavity; this may be called *præsciatic*. This form is distinguished from the preceding variety, not only by the difference in seat of the head of the femur, but also by the rotation of the limb outwards—a circumstance the more remarkable, that in all the other posterior luxations of the femur, the limb has been found to be turned inwards.

---

Memoir on the Present State of Naval and Military Surgery. Addressed to the Right Honorable Earl of Spencer, First Lord of the Admiralty. By *John Bell*. Yarmouth, January 20th, 1798.

We have been favoured by a friend with a copy of this Memorial of the celebrated John Bell, on a subject which is now attracting some attention. In another part of this Journal will be found a review of a pamphlet on the subject of Professorships of Military and Naval Surgery. Our reviewer, an accomplished army surgeon, has taken an unfavourable notice of the proposition, and his arguments are strong and powerfully urged. We have thought that it might be well to contrast with them the sentiments of Bell, that the public might compare the statements of both sides, and judge the more satisfactorily of their respective value.

With respect to the Memoir before us, it is scarcely necessary for us to point out the fire and energy which characterise it, or to dwell on that masculine vigour of style, which makes the writings of John Bell so eminently classic.—*Eds.*

My Lord,

Ever since I have been capable of thought, I have struggled for objects far beyond my reach to obtain. In this anxious moment, when I have about me no selfish views, no mean nor worldly cravings, no desires which I should not be proud to avow, there dwells upon my mind this oppressive and prophetic feeling, that I am perhaps going to suffer the sorest of all disappointments. I am upon the eve of either atchieving by my zeal a great public good, or of preventing, by an imprudent and too sudden movement, a new order of things in that department which needs most of all to be improved.

But, my Lord, I have a claim upon your Lordship, which you will never refuse. I have studied Naval Surgery with particular care. I have bestowed upon it, of money, of time, of labour, more than I am entitled to bestow. I have followed your victorious fleet, and attended your prisoners and your wounded, as if I had been attached to Government by old services and high rewards. These, my Lord, are my privileges. I am now retiring from this busy scene, and all my claim to your Lordship's notice is this desire to be useful.

I am but ill prepared to speak upon the most interesting of all subjects; I am fearful of that enthusiasm, which is so apt to mix itself with thoughts such as

I am now to expose to your Lordship; I am anxious, above all, to know how my advice may be received by those accustomed to judge of high matters:—yet still I feel myself entitled to propose the establishing of one great School of Military Surgery. It were almost, I think, an object of national gratitude; surely it were an institution humane, charitable, useful above all others; in my situation, I should feel what I now propose to be nothing presumptuous, nothing less than a sacred duty, which no fear of impropriety should repress. But when I perceive, by every movement, that it is the sincere wish of every man in power to raise this department to an honourable and distinguished rank—when their efforts are ineffectual only from the want of those suggestions which must come from medical men—I am no longer in doubt how my thoughts on such a subject will be received; and all those apprehensions which the nature of the occasion must excite, are lost in the exulting prospect of seeing a great national benefit grow out of this single thought, once vague and transient, but now rising in importance even while I am explaining myself to your Lordship. With such feelings labouring in my mind, and with an entire conviction that you will delight in whatever promises a public good, I must write with an enthusiasm in regard to the subject, and with a perfect freedom in all other respects, which your Lordship knows well how to excuse, perhaps to approve.

How shall I venture to tell you of the melancholy state into which the public service has fallen? It never was respectable, it is now disgraceful. Things are truly come to such a pass, that to point out the means of reformation must be a great relief. No plan of national education has ever been proposed. Every other branch of our profession is taught, apart and carefully, while Military Surgery, the most peculiar of all departments, has been left to chance; and in this line it is easy to trace the cause of many evils, too serious to be long thought of temperately or patiently. When a young man enters into the Navy, his education is but ill begun, and cannot improve. He is put down into a hole, there to remain for years. He is deprived of all communication, of all desire of knowledge. To breathe the vital air, he must live in the promiscuous conversation of a ward-room—Politics, History, Anecdote, News—everything is heard there but that which interests him most, which is the very business of his life. His youthful ambition is dead;—his profession! is forgotten; his first proud feelings, which sprung up with the first dawnings of knowledge, are buried there; his mind is vacant and powerless;—"and all his precious hours are running down to waste."

To the life of a navy surgeon there are, God knows, no seductions. Nothing, as it now stands, can drive a young man into such a service, but want of education, and want of friends; nothing can support him, even for a short term of years, through the difficulties and labours of this life, but a love of his profession, and a sense of duty above all obstacles—hardly, indeed, can any thing retain him in a service so poorly appointed, so little honoured; he never feels himself till he leaves it, returns to school, and begins his education anew!—and those pensions which Government has held out as permanent rewards, are but bribes for such young surgeons to continue their services only in the days of their ignorance, and to retire when they first become fit for service: thus do they complete their education with the accumulated wages of service which they could not perform. But behold, when their education is in some degree renewed, when they are fit to be received any where but in the fleet, they prefer the permanent establishment of village surgeon, in some remote and miserable place, to the service of the State; plainly declaring against that profession, to which they have given up the very prime and vigour of their life, a degree of contempt which, were it needful, I never should be able to express.

Be assured, my Lord, that while Government strives to attach men to its service—to lead them up to the knowledge of a difficult profession, through all the intricacies of a learned science, by money alone! it cannot succeed. Nor will men ever delight in a profession which is not made respectable, honorable, and useful. Men trained to the sea, find themselves entered into a way of life full of danger, but full of honour, and for this sake they love it. But ours is a profession where a thousand ways are open to ambition; every situation is easy, and gainful, compared with this of the Navy; and from year to year our fleets are thoroughly drained of all those whom Government wish to retain.

Indeed, my Lord, this is a serious business, and men willing to find, in all that has been done, nothing but negligence, profusion, and waste, will say, in derision, "Here now we see, how Government may, by a mean economy, ruin the most important of all establishments, and bring matters to such a pass, that young men of the lowest education, of the slenderest means, shall refuse the service;—daily advertisements shall be quite neglected;—examination shall fall into utter disuse, and all shall be promiscuously received;—the British seaman shall be more helpless in the day of battle, than the peasant when employed in his peaceful labours;—the poor man providing from his hard-earned pittance help for himself and family in the hour of illness, while the most dangerous service hardly extorts from such a government the very appearance of care."

Those, my Lord, are the reproaches to which a government rather uninstructed than careless is unhappily exposed; and it is all too true. Perhaps in a whole fleet there are few surgeon's mates, not one may be, who is able to perform the greater operations of surgery. It has happened that, often, after the most earnest entreaties of the officers, of the surgeon, of every one concerned, a-board; no, not one to screw a tourniquet, to tie an artery, to hold a shattered stump, to put a piece of lint to a bleeding wound!

These things, my Lord, must make a strong impression on the public mind, and must create very awful feelings in those who are concerned. Had those shots which have passed so often through the cock-pit, and which have killed so many among those who were already wounded, and who had retired to the place of help and safety! had one of those shots struck the surgeon—what must have been the condition of those who survived him! Inevitable death from wounds which are not deadly, is an awful sentence! who can bear it? Let the man of the most determined courage, my Lord, think but of this! and, if he have not that carelessness of life which deprives mere animal courage of all praise, let him say with what heart can he go into the midst of battle, where in a few moments all is horror, confusion, and dismay; where the danger of the hour makes no respect of persons; where the high and lowly are laid side by side, dead and dying! and the surgeon stands for a moment in his place,—alone—fixed and motionless,—with folded hands,—in horror and in deep astonishment at the situation in which he finds himself! "Can such things be, and you that behold them still preserve the natural ruby of your cheeks?"

Such are really the dangers to which that order of men are exposed, upon whom, more than ever in the annals of this empire, the very empire itself and all its future annals depends. I have, I hope, pressed these thoughts, as becomes me, not too rudely. Had the case been hopeless, I should have refrained; but while there is hope, I would bend with all the little power I have, on that lever which the weight of the state only can move. For this is indeed the truth: at one period of time we might have said "wars of conquest have surely ceased;" but we have lived to see conquest running her headlong course; overturning empires and states; and too well we know, that while we continue a maritime power, wars of commerce will never cease. We must maintain ourselves as a maritime power; this is the only means for internal safety in the present, and the only hope of our future success, even of our existence as a commercial state. Stations must be multiplied; new hospitals must be built; the whole establishment of our marine must be strengthened on every side; and nothing will give more splendour to the cares of Government, nor be more grateful to the public mind, than to see the Medical Department raised, improved, may I not say, created a-new.

Is it not proved, my Lord, "that the state cannot attach men to its service early in life; nor train them through all the intricacies of science by money alone!" and were it not easy to propose to young surgeons an object really worthy of their ambition, and a scheme of education which would make them delight in their profession, and incline them continually to improve? For a well conducted education is the highest bribe—to become the member of a great school, is itself a privilege—and to rise, by force of abilities and knowledge, to the head of such a school, were a noble reward. These hold out that kind of hope which supports a man through every great duty, whether of perseverance or sudden exertion; of thought or of bodily labour. Unless you can fix his hopes on some grand system of life, you will never obtain from a young man those services which, with high motives, he is able to perform. If you would ensure his diligence, endow him with knowledge.

The institutes of a National School are easily imagined. The object is expressly defined. The instruments by which it is to be accomplished are in daily use—and all its consequences are as sure almost as the operations of a mechanical power. Give the Professor all the means of instruction; draw out in due form the subjects of his lectures; the forms of teaching; the rules, the privileges, and the oaths of the pupils; and mark these things down as the institutes of the school.

I. The Professor must teach with perfect care the essentials of anatomy; the great principles of surgery he must found upon these dissections; and all the great operations, all the accidents, which each part of the body is liable to, all kinds of wounds must be fully explained.

II. These general principles of the science must next be applied to the peculiar duties of the military surgeon; the Professor must teach carefully the peculiar nature of gun-shot wounds.

III. He must deliver a short code of military medicine explaining the fluxes, fevers, spasms, infectious diseases, and all the peculiar duties of the camp and hospital, and he must explain the scurvy, ulcers, infections, and all the disorders most frequent in ships of war.

IV. He must teach medical geography—the climates, seasons, coasts of various countries. The manner of conducting soldiers on a foreign expedition—the general care of their health—the choice of encampments—the forming of hospitals on shore—how to attend an army in the field—how to convert churches, granaries, public buildings, into occasional hospitals—how to lay the wounded in besieged towns—how to carry them off the field in a retreating army.

V. Along with these, must be taught military œconomies, diet, general medicine, exercise, clothing, and all methods of preventing disease. Without this knowledge, no man is entitled to serve. How few are thus taught? How few are fit for service? How few are there who are not conscious of those blurs and blots in their general education, which no diligence of their own can ever do away?

VI. The last, and not the least important duty of the teacher, should be to point out for his pupils a future plan of study, to make for them a selection of books; to deliver critical and practical observations on those which are to be most used. The teacher must not only instruct his pupils for the present; he must select objects for their future study. He must teach them this truth, that their education is only begun, and that the best of their studies remain as yet untouched; he must show them how to think for themselves, and then he may hope to reap in his turn the fruit of their labours, and live to see their observations and cases published under his own care; he may, after a regular book of anatomy and surgery, see the whole enforced and illustrated by cases and observations; and, working along with his pupils, he may perpetuate the institution, by publishing the works of Military School.

To perform his duties with spirit and energy, his place must look like what it is; the centre of a Great School, which stands in an eminent and respectable station, whence well-instructed surgeons are continually going out on every line of service, and returning the inestimable benefit of a good education, with those kinds of knowledge for which they are well prepared; which they are continually collecting in all parts of the world; which they send willingly to that school with which they are still closely allied.

The Professor must in all respects have the perfect command of his school. He must have a Lecture-room, capable of being occasionally enlarged; he must have a Dissecting-room proportioned to so great a purpose, wisely conducted, no doubt, and modestly, but under the absolute protection of the law. He must have a Library, continually increasing, by certain fees, from occasional pupils. He must have a House of Assistants, like the clerks of a great hospital! one keeper of the library,—one a dissector for the class,—two to inspect the pupils' dissection—and two must be appointed to write, under the direction of the teacher, his lectures, his studies, the communications from his older pupils, and the extracts from the books. The character of so rich an education for those young men whom the teacher might prefer to such stations, would ensure them all kinds of promotion, and would be such a reward as money could not equal.

But especially, I say, he must always have a perfect command of his school! a Uniform for those who are of the school, and who are to have the first seats—his place surrounded with walls—his pupils must come gratis to that school, and must take an oath "to be faithful to their studies; to be serviceable to Government in every way; to be diligent in all the occasional duties committed to their charge; to employ their time in lectures, reading, dissections, till that period arrive in which they are to be appointed to service." Let every one who comes rather as a stranger to the military school, sit behind the regular pupils, and pay his fees to the institution, for the support of the library, and for defraying the general expense. Let those who, having once taken the oath, would leave the service, pay up, upon forsaking the school, all their arrears of fees, and put off that uniform which is the badge of their attachment to their country. Let all the school have the privilege of attending some great hospital, or rather give some great hospital the privilege of having those pupils attend its operations, (with a small fee,) for such an attendance would almost create an hospital. Let the library be open daily;—let there be fires in all the rooms;—let them be made comfortable for study, and a sort of home for those attached to the school;—let the great volumes of anatomical plates lie always on the tables;—let books be issued in circulation every week;—let there be duplicates, according to the judgment of the teacher, of every useful book.

Those who, about to enter into this way of life, would begin a regular education; and those who are at this moment engaged in service might, by a perpetual circulation, pass through this school, have their education renewed, and be assimilated with the whole. The school would be full in time of peace; it would send out its surgeons with increased industry and knowledge, when we had declared war. The pupils of this school would be examined by a board of control, where their teacher should have a seat, but no voice; where he could have no other interest but that of instructing young men, so as to answer like masters in their profession, even before they had begun to practise, and should be able almost to annul and render void the foolish formality of examinations, not by ignorance, but by superior knowledge.

The master of such a school would spontaneously perform other and higher duties; he would have, from his very office, such energy, so high a sense of his public duties, such versatility of talent, that, in all emergencies, he would render important service to the state. He would plan hospitals; would go to assist the sick and wounded; would help in the detail of the new establishments. Is it not distressing that, when a fleet has suffered in some severe engagement, Government has no general surgeon, whose advices and service they can command, that their wounded are abandoned to the carelessness of ignorant men, and shipwrecked, I may say, upon their native shores? Perhaps in certain seasons, he might sail with fleets, or accompany armies, from a desire of further knowledge. Perhaps he would give occasional lectures at the setting out of any great expedition, going down to meet with the surgeons of the fleet, to inquire about all their little wants—to see that they were indeed prepared at all points, to converse with them, to lecture to them. All these occasional duties he should do without expense to himself, that he might do them easily; and they should be voluntary, that he might perform them with zeal.

My Lord, I know too well that plans of national schools have been but the reveries of enthusiastic men, and, in this sense, I would almost avoid the name; yet, surely, an institution having such objects as these, so expressly limited in its purpose, so entirely practicable, so sure of attaining its end, stands in the first rank among plans of national education. This is, to a state, the only security it ever can have of procuring well-instructed and diligent men. Promotion would no longer go by courtesy. Examinations would cease to be an unmeaning form. Examiners, who are now afraid to touch those whom they are sure to find ignorant, (and yet dare not reject,) would then examine severely, because they would find knowledge to grapple with, and would be able to select well-informed young men.

It is now well known, that there can be no other security. We assure ourselves of a man's knowledge rather by his education, than by questions which are so often conned for the occasion, and repeated coldly, without interest, without understanding. No University trusts to this slippery test, but requires, even for ordinary studies, a peculiar and regular education continued for years. Certain courses are required absolutely, more general studies may be added to them; but those must be performed. In this sense a Military School should be a regular College. It should be erected in the centre of other schools. The pupils should have the choice of other teachers, and inducements to various and liberal studies. They should be early attached, and with sensible and sober views, to that service which now they fall into by chance and misfortune. They should be early trained to those parts of knowledge which now they learn from books, too late in their course of service to be of much use. They should be well read in those authors who now fall into their hands by a sort of accident. While they had their choice of other studies, they should feel themselves peculiarly responsible for their perfect acquaintance with all the lessons of the Military School. They should have, in short, every inducement to serve, but should feel no bondage in serving. They should be attached to the School only by the usefulness of their studies, but they should be allowed to leave it with no other forfeiture than that of losing their place and precedence.

The security is perfect also;—because, for the studies of such a shool, genius is not so highly necessary; diligence alone is required; in medicine and in surgery, above all! enterprise is dangerous, and experiment almost guilty.

That good sense which is so universally diffused, that plain knowledge which is so easily acquired, and that moderation which industry, and the knowledge of plain and simple matters always begets, are sufficient for all the occasions of life, and more to be prized, in my estimation, than the most splendid talents. How often is genius wild, ungovernable, dangerous? How much, on the other hand, do we value the marks of industry, humanity, modest knowledge, sure tokens of a useful man. And such, I am very sure, would be the character of every young man, who should issue from such a school.

It is true,—that in every great institution connected with a state, the Means also must be planned. But, my Lord, I am well assured, that this institution needs only to be put in motion; that a man, capable of arranging and conducting such a School, might make himself independent of all external aid; and (should Government be blind to so high an interest) could support himself in every thing, but the splendour which attends the forms of a National School. For this plan of education belongs to one-half of those bred to our profession, since more than one-half of those educated to medicine in the common schools belong to the Navy, the Army, the India Department, or our foreign dominions; and although, perhaps, the India department might not directly contribute towards the establishing of such a school, yet where lectures were given on anatomy, and surgery, and naval medicine, and climates, and contagions, and all the accidents and adventures to which foreign troops are exposed, all the surgeons of the India department would become voluntary Cadets of that school, where alone they could learn their duties. Pupils not attached to Government would come to it from every department,—from all parts of the kingdom—perhaps from other countries. Their fees, given to the secretary of the institution, would defray much of the expense. The Library of itself would grow out of small fees, as every library must do. The Museum belonging to the school would be made in the school; the books which might be published as the works of the school would also be a source of gain. Every movement of such a machine should strengthen its powers.

In Mechanics, a great Engine wears out the very materials of which it is composed; but in Politics, the materials of a great institution like this, are in our hands: we have but to arrange them so as to begin the motion! and while the movement goes on, it draws into itself every thing which belongs to it, and becomes a great system, self-improving, and continually progressive, begetting an enthusiasm in all who are allied to it which supports the whole.

Even should it be apparently expensive, that expense would be repaid a hundred-fold; for ŒCONOMY OF LIVES IS THE GREATEST ŒCONOMY TO A STATE. And, indeed, we must have this ungracious thought still in our mind, that each sailor and soldier is but a living animal, strong and active, purchased for a certain use. Each costs Government a sum of money equal to the one-half of a surgeon's annual income. Then counting them but as living creatures, bought for a certain use, if in a year the surgeon saves, by his superior knowledge, but a few lives, he repays Government much more than that small sum which in the beginning of the year Government, I may say, had lent him; and this debt is so fairly returned by every improvement in the care of lives, that I am very sure the professor of such a school, and each of his pupils in his proper place, would repay the bounty of the state a hundred-fold. Nothing would more strengthen the hand of power than a general establishment, which were at once grand and commanding in its aspect, and in its nature humane and useful.

Indeed, such an institution, under any government, and by all parties, should be praised and cherished. It would be to the state a saving of lives infinitely desirable—it would do away a flagrant reproach;—it would become to the public a rich property, which the simple idea itself would bestow almost without expense—it would be a fair succession and inheritance—an object of high ambition to all the surgeons attached to the service of the state. Then our ships would not go into battle without well-instructed Surgeons, and Mates capable of every operation; nor would low fevers, almost equal to the plague, rage unopposed; the surgeons of such a school would never shrink from any service of danger; our troops would no longer die in warm climates, by thousands, uncared for, and almost careless of life! What should we not save in lives, and in money the price of lives?

But you must be delighted most of all, my Lord, with the sentiments which must arise in those who remained faithful disciples of such a school. While learning, they would become enthusiasts; when advanced to higher duties, they would be worthy of being supported in a rank becoming their education; when advanced to years of experience, and well trained in those studies which their teacher had begun—they would look forward to the most honorable of all the appointments which Government could bestow on professional men, the Teaching of the School itself. And those few who, in the course of years, from various connexions, and the thousand accidents of life, fell off into the line of private practice, would be valued in proportion to the severe education and honorable conduct of that school in which they were bred. They would be more honoured then, than they are neglected now. They would be like the children of a careful father, who said, "My sons, I have little to bestow, but you have been bred up like brothers in industry, charity, knowledge, and public virtue. These are to be your comforts, your pride, your distinction in society; and for your rewards; I hope you will so conduct yourselves, that you may look up with confidence to the gratitude of your country."

My Lord,—if I have a fear for the success of this plan, perhaps it is that of an enthusiastic but honest mind, that it will be too useful, too commanding to be endured! for even here—there is some danger in Greatness. On the first view of a plan in which other institutions will be absorbed, lesser offices annihilated, and great appointments comprehended within its sweeping circumference! what will not little men say? There is indeed one duty which I owe to this institution, which I fear I shall never perform; there is, I know, a sort of winning policy, a time-serving and flattering spirit which such movements require; there is needed infinite cunning and management, not of great objects, but of little minds.

But, my Lord, I am not a politician either by breeding or by nature. I would not strive against little arts; I hardly know the ways; I should hardly condescend to use them; I would not work upwards through interest, cabal, and petty solicitations to your Lordship's favour; I claim your protection not for myself, but for a great scheme which embraces a great public good; I would have it operate with that independent influence which becomes a great establishment, downwards from the higher powers, through all the lesser objects of arrangement, economy, policy, and energy within itself.

Once more, my Lord, allow me to express a degree of anxiety to know "how this is to be received by those accustomed to judge of high matters." Let it stand or fall by itself; unconnected with me, or my little purposes, for such will be supposed—"let it be given to the wisest," my whole desire is that it may be useful. Whoever may be appointed to fulfil this plan, I shall assist him with advice, with books, with manuscripts, with drawings, with plans, heartily and honestly; without irritation, without envy, without reserve; of which promise, let this be my solemn and public pledge. It is the fortune of those who pursue medicine as an art only, to become rich, but without honor. It is the fate of those who attach themselves to science alone, to struggle with difficulties, and this, my Lord, is my condition! But such honorable difficulties I would not forsake for the ease and affluence of richer men. This was once a passion, but now it is a fixed habit of mind, in me, a second nature—indeed, I am not building a ladder for myself to climb upon to some ambitious height; I am thinking, believe me, my Lord, more of others than of myself. I am not fit, nor willing to be, removed from that spot where chance has rooted me. Everything rests with your Lordship. This plan is unencumbered with little designs on my part; it is submitted to your Lordship, because in so responsible and so high a station, you are, as an organ of the public mind, to judge betwixt me and that public, what degree of notice these suggestions deserve.

This is in every sense a private communication; no other copies have gone out of my hands; no great man has been solicited; no private friend has revised it; it is printed only for your Lordship's use, and to do justice to the subject; —and will you, my Lord, also condescend to receive it in this form as a mark of particular respect. JOHN BELL.

Yarmouth, January 20th, 1798.

---

### Remarks on Gonorrhœa in the Female; on the Transmission of the Inflammation from the Uterus to the Peritonæum by the Fallopian Tubes; and on the Sterility which is the Result. By Dr. *Mercier.*

Several papers have lately been published in the French journals on the subject of the seat of gonorrhœa in the female. According to M. Gibert the favoured spot is the meatus urinarius; in 116 cases, he says, he has constantly remarked, in the first weeks of the disease, a catarrhal suppuration of the urethra; he adds, moreover, that vaginitis is rare, and that out of several hundred women whom he has examined with the speculum, he has not met with a well-marked discharge from the vagina in more than five or six. Others, however, do not appear to have been quite so unsuccessful; Ricord, after stating that he has been surprised at the frequency of the urethral discharge, which he has seen eight times at least in twelve, adds that, in the majority of cases, the affection of the vagina is more prominent than that of the urethra. M. Delmas agrees with Ricord; and M. Durand-Fardel, though he has never met with urethritis without discharge from the vulva or vagina, has often the last without the urethritis.

According to Dr. Mercier, the inflammation always commences in the mucous membrane of the vulva; the patients experiencing there a troublesome itching increased by walking, or the introduction of injection-tubes. The labia minora are usually red and swollen, and their condition easily accounts for the pain experienced in a certain number of cases on making water. After the lapse of a short period the inflammation extends to the neighbouring canals, the urethra and the vagina, and from the vagina it may extend to the mucous membrane of the uterus.

Dr. Mercier compares the course of gonorrhœa in the two sexes; in man, he says, it usually begins in the meatus urinarius, and then passes more or less deeply. If arrested before reaching the more distant parts of the canal, it first quits the parts last attacked, is limited to its original seat, and finally disappears. If, however, it gains the neck of the bladder, the case is altered; the inflammation of the neck of the bladder, and the neighbouring parts of the urethra is *très-tenace*, either on account of their functions, or of their structure, and especially on account of the presence of the prostate gland. The inflammation may then often be seen to disappear in the middle portion of the urethra, whilst it persists at the two extremities. A similar state of things, according to M. Mercier, often exists in the female organs; the inflammation occupies the entrance of the vagina and all the mucous lining of the urethra, whilst but few traces of it can be found in the intermediate parts.

If this statement be correct, there can be no difficulty in conceiving that, in the same way as inflammation of the urethra often extends to the testicles by the spermatic cord, so the inflammation of the mucous membrane of the uterus may be transmitted by the Fallopian tubes to the peritoneum. In illustration of this, Dr. Mercier relates a case of peritonitis occurring in a patient of his affected with gonorrhœa; on examination after death, the external organs of generation were found inflamed, the inflammation extending up the vagina, where it was less marked, the mucous membrane of the uterus and Fallopian tubes was of a deep red colour, and covered with puriform mucus. The peritoneum about the broad ligaments was much inflamed, and presented soft reddish false membranes, especially near the fimbriated extremities of the Fallopian tubes; in other parts is was healthy. In this case, Dr. Mercier is of opinion that the inflammation extended from the labia to the peritoneum.

It is not uncommon, he remarks, to find traces of inflammation pretty exactly limited to the free extremities of the tubes, both of which often present in the same female false membranes, without the existence of any in the intermediate parts. In a case which he had the opportunity of examining, in which a polypus existed in the uterus, the mucous lining of this organ was found red and inflamed, and the peritoneal extremity of the Fallopian tube was seen to be adherent to the neighbouring organs. Hence he concludes that every inflammation of this membrane may be transmitted to the peritoneum by the tubes, and determine, at the point where the mucous membrane of the genital organs is continuous with the serous membrane of the abdomen, the production of false membranes. If there result an obliteration of both fimbriated extremities, sterility is the necessary consequence; but if, on the other hand, these false membranes only cause the fragments to adhere to the neighbouring parts, and prevent them from being inclined towards the ovary to receive the germ, extra-uterine pregnancy may occur.—*Revue Médicale.*

---

### Diseases of the Lungs from Mechanical Causes; and Inquiries into the Condition of the Artizans exposed to the Inhalation of Dust. By *G. Calvert Holland*, Esq. M.D.

In a notice in a late number of this Review (No. 78, p. 463), of a little work, "The Vital Statistics of Sheffield," by the same author, we spoke of the injurious effects produced on the health by the inhalation of the gritty and metallic particles evolved in the process of grinding; we shall therefore content ourselves at present with an account of a plan, certainly simple, and which is said to be equal to the thorough correction of the evil.

"A wooden funnel, from ten to twelve inches square, is placed a little above the surface of the revolving stone, on the side the farthest from the grinder, and this funnel terminates in a channel immediately under the surface of the floor; *or we may consider the channel simply as the continuation of the funnel,* in order to avoid any confusion in the explanation. The channel varies in length, according to the situation of the grinder, in reference to the point where it is most convenient to get quit of the dust. If we suppose that eight or ten grinders work in the same room, each has his own funnel and channel, *and they all terminate in one common channel,* the *capacity of which is perhaps twice or three times as great as each of the subordinate or branch channels.* The point where they terminate is always close to an external wall. At this point, within the general channel, a fan is placed, somewhat in form like that used in winnowing corn, and to this is attached a strap which passes upwards and over a pulley, so that whatever puts the pulley in motion, causes the fan also to revolve. The pulley is placed in connexion with the machinery which turns the stone, so that whenever the grinder adjusts his machinery to work, he necessarily sets the pulley and the fan in motion. The fan, acting at this point, whatever may be the length of any of the subordinate channels, causes a strong current to flow from the mouth of each funnel, which carries along with it all the gritty and metallic particles evolved, leaving the room in which the operations are pursued, free from any perceptible dust. When the whole apparatus is perfect and in excellent condition, the atmosphere of the place is almost as healthy as that of a drawing-room."

The expense of the construction of the apparatus, it is added, would scarcely exceed the proportion of a sovereign to each grinder. It is better that the subordinate channels should be *under* the floor, and not *above,* as they are then less liable to accidents. Dr. Holland states that, where this contrivance has been in operation for years, he has not found a single individual labouring under any pulmonary affection. He recommends the Legislature to interfere, and make it imperative on the part of the proprietor of wheels, to construct such an apparatus, and compel them to keep it in a perfect condition.

Dr. Holland deserves great credit for his exertions on behalf of the "needy knife-grinders."

### Galen on the Hand.

This little brochure is without the name of either author or publisher, and is probably a TENTAMEN MEDICUM, or written and printed for amusement and private circulation. It is curious, as shewing, for the ten-thousandth time, that there is nothing new under the sun. All Charles Bell's ingenious observations on the *design* of the Almighty Architect in the construction of the human hand, have been anticipated, and more than anticipated, by the old Greek Physician and Physiologist. Galen and Bell, however, might have employed the same arguments and illustrations in the examination of every part of the human frame —nay, of every animal and vegetable structure on the surface of the earth, and in the waters under the earth. Where can we look around, above, or below, without seeing and acknowledging the "works of an Almighty hand." MAN, it is true, as the last and most perfect piece of mechanism, offers the most obvious and striking proofs of benevolent design and consummate skill. Man has not the swiftness of the horse—the teeth, claws, or power of the lion—the horns of the bull—the tusks of the boar—the might, the destructive jaws, and the almost impenetrable hide of the crocodile. He is born naked and defenceless; but he has a brain to devise, and a hand to execute all kinds of weapons, snares, and armour, by which he subdues the most ferocious animal that treads the earth—brings down the feathered tribes that wing the air—and hauls up on dry land the monsters of the deep for his use or sustenance. But, although the human hand is admirably adapted for the construction of everything that is useful, ornamental and destructive, it is only an instrument, after all, and would never have given him the dominion over other animals, had it not been for REASON, by which he is distinguished from the other inhabitants of the globe. Galen observes, in opposition to Anaxagoras, that man has a hand *because* he is the wisest of animals—and not that he is the wisest, *because* he has a hand. The monkey has a hand nearly as well adapted for manipulation as that of the human species; but he has not the brain to give that hand its full scope of action. The following extract may afford a sample of the close reasoning of the Old Greek.

"It appears to be the best constructed of all prehensile organs. Forasmuch as the hand can form a circle round a sphere, grasping it on every side, it also as securely and firmly holds the straight and concave; which, if it be so, it can grasp all forms, for they are all formed from three figures, convex, concave, and straight. But, since many bodies are too bulky for one hand, nature has given a second, an auxiliary to the other: that each grasping opposite sides, should not hold it less securely than one very large hand. For this reason they are placed opposite each other (for they are formed for mutual use), and are in every respect equal: for they are the same organs, and have similar duties. Consider the largest body a man can grasp with both hands, as a tree, or a stone; and again, the smallest thing perceptible, as a grain, a hair, or a thorn; and then how great a number of bodies intervening between the largest and the smallest; you will find man grasping all these, as if the hand had been formed for each. Man seizes the least bodies with the tips of two fingers, the index and the thumb, (which we Greeks call megan,) and bodies a little larger with the thumb and the same finger, but not with the tips; for bodies still larger he employs three, viz. the fore, the middle, and the thumb; and if the body be still greater, he uses three fingers and the thumb, then all four with the thumb; afterwards he seizes with the whole hand; and finally he seizes with both hands. It would have been impossible to perform any of these actions, if the hand had not been divided into variously formed fingers. Nor is it sufficient they should be simply divided; for suppose the thumb had not been placed opposite the four fingers, but all five placed in the same line, is it not plain the number would have been useless? For to grasp securely, it is necessary either that the whole body be encircled on every side, or wholly grasped on the two opposite sides. This power could not have existed, if all the fingers had been placed in the same straight line."

But let not MAN be too proud of his brain and his hand! If these have invented and constructed so many implements and articles for the protection, comfort, and luxury of his species, they have not been inactive in evil doings. The same hand that elaborated the power-loom and the steam-engine, founded also the cannon and the cutlass for the destruction of human beings! The same hand that wields the pen in the cause of religion, morality, science and literature, directs the same instrument in the dissemination of Atheism, vice, and sedition. The same hand that forged the ploughshare and spade to cultivate the soil and call forth bread, forged also the manacles and chains by which the SLAVE is dragged from his native land, and imprisoned in the dark Peruvian or Siberian mine—or scorched beneath a tropical sun in hopeless captivity! In fine, it is very doubtful whether MAN has more reason to be proud than ashamed of his boasted hand.

---

### Mémoire sur l'Emploi du Carbonate d'Ammoniaque dans la Scarlatine, &c. Par le *Docteur Rieken*.

Dr. Rieken on the Employment of Carbonate of Ammonia in Scarlatina.

DR. RIEKEN is a Belgian physician of very considerable reputation, and appears to have seen a great deal of the disease on which he writes. Although the cases in which he has himself employed carbonate of ammonia and other ammoniacal preparations are not very numerous, yet, as extracts are given from the works of most authors of all countries who have made use of this medicine, the work is one of much interest, and the conclusions drawn of considerable value.

It would appear that there have been some epidemics of scarlatina in which the administration of ammonia, joined to a suitable hygienic regimen, has proved of the greatest service. Such were the epidemics, apparently of a malignant form, mentioned by Peart, Strahl, and Bodenius. At the same time, it must be borne in mind, that Bodenius attaches much importance to the maintenance of pure air in the chamber of the patient, sponging with vinegar, &c. In other epidemics of scarlatina, such as that observed by the German physicians at St. Petersburgh, ammonia was employed without success, or at least without any remarkable degree of success.

It is in that form of scarlatina, called nervous, that the employment of carbonate of ammonia, according to the observations of Roesch, Stoeber, and of Schlesier, has been followed by the most happy results. In inflammatory scarlatina, and especially in that which is accompanied by cerebral symptoms, carbonate of ammonia is of considerable service; but its use should be preceded or accompanied by the employment of a more or less energetic antiphlogistic treatment, such as bleeding, leeches, neutral and purgative salts, and especially calomel, cold fomentations and lotions, &c. In the gastric form of scarlatina, the internal administration of ammonia has not been attended with much benefit, but it seems frequently to contribute to the cure of the patients when used in the form of lotion.

Withering and Schlesier consider that gargles, into the composition of which liquid ammonia enters, act to a certain extent as a prophylactic against scarlatina; frictions also have been found useful in the consecutive dropsy as well as in the secondary swellings of the parotids.

The dose of the medicine varies, of course, according to the age of the child. Dr. Rieken recommends that from one to two drachms of carbonate of ammonia should be dissolved in six ounces of distilled water, with a little syrup. Of this form one to four drachms may be given every hour or every second hour, according to the urgency of the case.

The remedy should not be administered after all imminent danger has disappeared. The immoderate and prolonged employment of it may give rise to very serious consequences. Mitscherlich, who has had considerable experience as to the effects of these salts upon the animal organism, states that all the preparations of ammonia employed internally form in the stomach and in the small intestines a great quantity of mucosities. The preparations are absorbed, and from this absorption there results a change in the blood, which consists in the blood coagulating much more slowly and in less quantity than in the natural condition.

These preparations have a specific effect upon the small intestines. In all the experiments in which ammoniacal preparations were introduced into wounds, but little change was discovered in the stomach, whilst the small intestines were much affected. The duodenum and the lower portion of these intestines presented less change than the upper and middle portions. This alteration of structure was joined to a considerable accumulation of blood in the vessels. The peristaltic movement of the intestinal canal was never abolished even in those cases in which the destruction of the intestines was very considerable; though in the cases of poisoning produced by salts of lead and copper, the peristaltic action is altogether annihilated. Hence M. Mitscherlich concludes that the muscles and nerves of the intestinal canal are in no degree interrupted in their functions by these salts; the cause of death must be sought for in some change in the composition of the blood.

With regard to the properties of carbonate of ammonia, to which the salutary effects of this medicine are to be attributed, much difference of opinion exists; the hypothesis adopted by our author is that of Bodenius, who considers that carbonate of ammonia acts partly by its fixed, partly by its volatile principles. The first, when employed in continued doses, enters into the mass of the blood and ameliorates its *crasis;* whilst the volatile principle removes the state of depression existing in the nervous system. The reasons advanced in favour of this explanation by our author, are as nearly as possible these.

1. The symptoms by which scarlatina, especially the malignant form, is accompanied, the frightful quickness with which it often destroys life, and the rapidity with which the decomposition of the dead body takes place, all these circumstances are in accordance with the opinion advanced by Bodenius and others; viz. that there exists in scarlatina an intoxication of the blood, and that the action of this poisoned blood upon the brain and nervous system, produces the symptoms of scarlatina, especially of the malignant kind.

2. The experiments made by Heidenreich induce us to believe that, in scarlatina, a free alkali is in fact eliminated from the body and deposited upon the skin. It is moreover probable that this alkali is of an ammoniacal nature, since Heim has found the odour of the perspiration of scarlatina patients analogous to that which is exhaled from old cellars, and Wendt does not hesitate to say that this perspiration gives out an ammoniacal odour.

3. The experiments of Mitscherlich prove that ammoniacal preparations produce changes in the condition of the blood; and this, acting upon the brain and spinal marrow, causes, according to this author, changes in these organs which, though unknown at present, may occasion death.

4. Now if there are in scarlatina changes in the condition of the blood which act in an injurious manner upon the brain and nervous system; if these changes consist in the fact that, by the introduction of the scarlatina virus, an alteration is effected in the ammonia which is found in the normal condition in the body and especially in the blood of man, and that this ammonia is even in part eliminated from the body in scarlatina; if it is moreover proved that ammonia produces changes in the blood which again cause other changes in the brain and spinal marrow; it cannot be denied that there is no medicine more fit than ammonia as well to restore to the blood the ammoniacal particles which it had lost in consequence of the scarlatinous affection, as to remove the consequences injurious to life, which are the result.

Such is the reasoning of Dr. Rieken, ingenious rather than conclusive. In this country did we give ammonia at all, it would be in the typhoid form of the disease. Depletion is always dangerous.

---

### ON THE PARASITIC VEGETABLE STRUCTURES FOUND GROWING IN LIVING ANIMALS. By *John Hughes Bennett*, M.D. Edinburgh.

IN the Comptes Rendus des Séances de l'Academie des Sciences for July and August, 1841, M. Gruby has inserted some observations regarding the crusts of *Tinea favosa*, or *Porrigo lupinosa* according to Bateman, for the purpose of establishing a more complete diagnosis of this disease than had previously existed. For this purpose, he had recourse to the microscope. By means of this instrument he ascertained—1st, that *Tinea* consists in the aggregation of millions of mycodermatous plants. These are formed of articulated filaments of a diameter from $\frac{1}{1000}$ to $\frac{1}{250}$ of a millimetre; they spring from an amorphous mass of which the periphery of each capsule of tinea is composed, and give off towards its centre oblong or round homogeneous corpuscles, which are the reproductive spores. The longitudinal diameter of these corpuscles is from $\frac{1}{300}$ to $\frac{1}{100}$ of a millimetre, and the transverse is from $\frac{1}{300}$ to $\frac{1}{150}$. The cells of the tubes sometimes contain small round transparent molecules, of a diameter varying from $\frac{1}{10000}$ to $\frac{1}{1000}$ of a millimetre. The seat of these vegetations he ascertained to be in the cells of the epidermis. The true skin is compressed, not destroyed; and the bulbs and roots of the hairs are only secondarily affected. The disc of the capsule, which is not at the commencement perforated, opens by a small hole in the centre. This enlarges, and the plants push through it, so that, at a more advanced period, instead of there being a central depression in the capsule, there is a convexity, and its edges disappear. M. Gruby inoculated 30 phanerogamous plants, 24 silk-worms, 6 reptiles, 4 birds, and 8 mammifera, but only induced the disease once, and then in a plant. The human arm was inoculated five times, but, independent of a slight inflammation and suppuration, no effect was produced. Dr. Bennett some time after examined the crusts on the head of a boy who laboured under the disease, and immediately detected the cylindrical and ramified appearances described by M. Gruby. Dr. Bennett, on continuing his observations still further, satisfied himself that pustules are not essential to the disease, though frequently present. Hence will appear the error of classifying the *Porrigo lupinosa* among the Pustulæ. According to Dr. Bennett, desquamation of the cuticle always precedes the development of the disease. Dr. Bennett made several observations in order to ascertain the correctness of M. Gruby's statement, viz. that the plants grow in the substance of the epidermis. He found that the entire inferior surface of the capsule is formed of epidermic scales, thickly matted together. These are lined by an amorphous, finely-granulated matter, from which the plants appear to spring. Superiorly, however, the epidermic scales are not so dense. These observations indicate the probable mode in which these plants are deposited on the scalp. We have seen that the appearance of the peculiar porrigo capsule was preceded by a desquamation of the cuticle. Hence it is more probable, that the matters, from which the vegetations are developed, insinuate themselves between the crevices, and under the portion of epidermis thus partially separated, than that they spring up originally below, or in the substance of the cuticle. Dr. Bennett failed, as did also M. Gruby, in communicating the disease to other individuals, or from one part of the same individual to another, although it is generally conceived to be of a highly contagious nature.

Dr. Bennett mentions that he has observed crusts upon the face of a living common house mouse, similar in every respect to those which constitute the Porrigo favosa in man. The crusts were of a more irregular form, prominent in the centre, not forming distinct capsules or perforated by a hair. When examined microscopically, they presented the cylindrical tubes and sporules *en masse*, in every respect identical to those which grow on the scalp of man. It has been observed that the odour of the crusts of Porrigo favosa is similar to that of mice, which makes it not a little singular, that the mycodermatous plant, constituting this disease, should be found growing on these animals. Whether the disease be peculiar to Man and the Rodentia, is a question yet to be answered.

*Cryptogamic Plant found growing in the Sputa and Lungs of a Man who laboured under Pneumo-thorax.*

Dr. Bennett, in making microscopic examinations of tubercle, tuberculous sputa, and the lining membrane, observed on some occasions fragments of tubes, more or less matted together, which appeared distinctly jointed, and which led him to suppose that a vegetable structure must occasionally be developed in the matter of tubercle found in the lungs. This supposition he afterwards ascertained to be well-founded. On examining the sputa of a man in the Royal Infirmary, the most beautiful and regular vegetable structure was observed. The individual was labouring under phthisis in its last stage, with pneumo-thorax. On examining a drop of the inspissated purulent-looking matter, discharged by expectoration, with a magnifying power of 300 diameters, he could perceive long tubes, joined at regular intervals, and giving off several branches. They varied in diameter from $\frac{1}{100}$ to $\frac{1}{200}$ of a millimetre, and appeared to spring without any root from an amorphous soft mass. Interspersed amid these tubes were numerous round and oval globules, often $\frac{1}{75}$ but generally $\frac{1}{100}$ of a millimetre in diameter, which here and there assumed the form of bead-like rows. Both the jointed filaments and sporules were developed in great abundance on the sides of the spit-box containing the man's sputa, which, in this situation, were inspissated, and presented a yellowish coherent and viscous layer. Two days after, the man died, and the left lung was found studded with cavities of different sizes, some of which communicated, by fistulous openings, with the cavity of the pleura. Several of the smaller cavities were partly filled with soft tuberculous matter. On examining this matter microscopically thirty-six hours after death, exactly the same appearances presented themselves as above-described. Numerous jointed transparent tubes, were readily observed, mingled with round or oval corpuscles. Dr. Bennett has no doubt that these vegetations existed in the man's lungs during life; *first*, because they were apparent in sputa freshly expectorated, and 2dly, because they could not have reached such a state of development, as has been described, in thirty-six hours. They continued to grow and develop themselves in the tubercular matter, after the removal of the lungs from the body, as well as in the matter discharged before death by expectoration. They resembled the Penicillum glaucum of Link.

*Crytogamous Plant found growing on the Skin of the Gold Fish* (*Cyprinus auratus.*)

Mr. Goodsir first examined with the microscope the vegetations found growing on the gold-fish. The fish in question was observed to be in a languishing state some time before death, and to be covered with a white efflorescence, of considerable length, which sprung chiefly from the dorsal fins and tail, and floated in the water. Some days after death Dr. Bennett examined some of the filaments microscopically. When viewed with a power of 300 diameters, two distinct structures were observed; one of them might be called cellular, the other non-cellular. The cellular structure was composed of elongated cells, which varied in thickness from $\frac{1}{100}$ to $\frac{1}{50}$ of a millimetre, presenting the appearance of long-jointed tubes. They were frequently branched, generally dichotomously. Some of the cells were empty, and seemed very transparent; others were full of granules. In most of the cells a distinct nucleus existed. Some contained two nuclei. The nuclei were generally placed at the proximal end of the cell, from which came off sometimes two other cells, more rarely three.

With respect to the substance from which this jointed structure arose, it seemed to be an amorphous mass, composed of very minute granules almost identical with the matter found in the capsules of the Porrigo, and tubercular cavities formerly described.

*Facts observed by various Authors connected with the Growth of Parasitic Vegetables in Living Animals.*

Laurent* has observed cryptogamous vegetations in the eggs of the *Limax agrestis,* which more or less impede the development of the embryo. These vegetations arise generally from the walls of the internal tunic of the egg, ramify in the albumen, and form in it a net-work which is sometimes checked by a vigorous embryo; there is here a struggle between the vegetable and animal development. The vegetable filaments may also be seen to arise from the body of a dead embryo, or of a non-developed vitellus. *Ledermuller* noticed the fact, that on leaving dead flies in water for a certain time, plants spring from the surface of their bodies. Similar observations have been made by several others. Parasitic vegetables have also been observed on living insects. On this subject see Kirby and Spence's Entomology, Vol. iv. p. 207. The disease in silk-worms, named *Muscardine,* is characterized by the appearance after death of a white eruption on the body of the animal, which has been shown to consist of numerous cryptogamous plants. In Valentin's Repertorium, it is stated that Ehrenberg has found *Chætophora (Tremella) meteorica* growing on the scales of the *Salmo eperlanus.* Owen†, on dissecting a flamingo, observed a green vegetable mould or mucor, growing on the lining membrane of tubercular cavities in the lungs, and on that of the smaller ramification of the bronchial tubes. Thus, he says, *Entophyta* exist in animals, as well as *Entozoa.* ENDES DESLONGCHAMPS noticed a similar appearance growing in an albuminous layer, which was effused on the membranous lining of the air-passages in an eider-duck. SERRURIER and ROUSSEAU mention having seen a vegetable mould in a hind (*Cervus Axis*). With this exception, and that of the mouse above alluded to, parasitic vegetations amongst the mammalia have hitherto only been found growing in man. Schönlein of Berlin was the first to recognise and figure a vegetable structure in the crusts formed on the scalp, in the disease named *Porrigo lupinosa,* by Bateman. Gruby of Vienna has given the most perfect description of these vegetations. Langenbeck observed a high degree of fungous development in the body of a man who died of typhus. It extended from the amygdalæ and upper part of pharynx through the œsophagus to the cardia. Meynier, of Orleans, has put forth the opinion that warts in man are true *gymnosporanges,* and that other human diseases are equally due to the development of cryptogamous diseases. Eschricht, of Copenhagen, states that vegetations sometimes exist in aphthæ.

We shall now state the *conclusions deduced from* the facts just detailed. The first is, "that these vegetations always arise in living animals previously diseased; 2nd, that their presence indicates great depression of the vital powers and impairment of the nutritive functions; 3rd, that the peculiar constitution favourable to their growth is the tubercular or scrofulous in the mammalia, birds, or fishes, and most probably in reptiles and insects: and 4th, that the therapeutic

---

* L'Institut, Tom. vii. p. 229.

† Philosophical Magazine, 1833. Vol. ii. p. 71.

indications are—1, to invigorate the system, and 2, to apply locally such applications as tend to destroy vegetable life. Dr. Bennett has found more than once a vegetable structure in the black deposit, which collects on the teeth and gums of individuals in the last stage of typhus."—(*Transactions of the Royal Society of Edinburgh*, Vol. XV., Part II.)

---

### Pathological and Histological Researches on Inflammation of the Nervous Centres. By *John Hughes Bennett*, M.D.

Dr. Bennett states that, for the last twelve months, he has neglected no opportunity that presented itself of examining microscopically the brain and spinal canal. Some of the results of his researches are recorded in this little work. Thirty-three cases are related in which softening of some part of the brain or spinal cord was discovered; these cases are highly interesting in themselves, and rendered still more so by the valuable and practical remarks appended to most of them. It is, however, to the conclusions at which Dr. Bennett arrives that we must confine ourselves in this notice.

### On the Nature of Softenings of the Nervous Centres.

Much uncertainty exists with regard to the nature of the softenings so often met with. Dr. Bennett is of opinion that two kinds of cerebral and spinal softening exist, an inflammatory and a non-inflammatory, which may always be distinguished from each other by means of the microscope.

1. *Character of Inflammatory Softening.*—On examining under the microscope a portion of inflamed and softened nervous tissue, in addition to the normal, tubular, and granular structure, there will be found, 1st, exudation granules coating the vessels, or floating loose, either isolated or in the form of masses; 2dly, exudation corpuscles, with distinct cell-walls, and sometimes nucleated. The more pultaceous and diffluent the softening is, the more numerous are the granules and corpuscles. The nervous tubes and normal structures also then become more and more broken down. With regard to the *nature* of inflammatory softening, it appears to result from the active growth, development, and breaking-down of nucleated cells (exudation corpuscles) in the effused blood plasma. "It is not a mere maceration of the textures in serum. No doubt the serum performs an essential part in the process, inasmuch as moisture is necessary for every species of growth. But we are of opinion that softening cannot be considered as dependent on inflammation without the existence of these bodies. So far from being connected, as some have supposed, with diminished nutrition, it is, in point of fact, an increased nutrition in the excess of blood plasma effused."

2. *Character of Non-inflammatory Softening.*—Here the cylindrical and varicose tubes of the part are found more soft and easily separable from each other. They have more or less lost their natural firmness and consistence; are readily torn across; the varicosities are easily enlarged by pressure, and, when separated or broken off, assume a globular form. The tubes also are more or less broken down. No exudation granules, masses or corpuscles can be detected.

The causes of non-inflammatory softening are four in number: 1st, mechanical violence in exposing the nervous centres; 2nd, a mechanical breaking up of the nervous tissue, by hæmorrhagic extravasations, either in mass or infiltrated in small isolated points, constituting capillary apoplexy; 3rd, the mere imbibition of effused serum, which loosens the connexion between the nervous tubes, and diminishes the consistence of the nervous tissue; 4th, the process of putrefaction.

Some authors have endeavoured to distinguish inflammatory from non-inflammatory softening, by the presence in the former of a zone of red vessels, or of purulent matter; this distinction, however, according to Bennett, is not a valid one, inasmuch as, according to his observations, the zone of red vessels is very rarely met with in inflammatory softening, and the infiltration of purulent matter has no real existence. The opinions which attribute softening to a lesion *sui generis,* to diminution of nutrition, to gangrene, obstruction of arteries, &c. are also considered hypothetical in the highest degree.

The symptoms which accompany the two forms of softening differ widely. In twenty-four observations, in which cerebral softening was discovered, exudation corpuscles existed in eighteen, in the other six no traces of these bodies could be found. In four, however, out of the eighteen cases of inflammatory softening, there also existed in another part of the brain non-inflammatory softening. In the fourteen cases of simple inflammatory softening, well-marked symptoms invariably existed, such as loss of consciousness, preceded or followed by dulness of intellect, contraction and rigidity of the extremities, or paralysis.

In three of the six cases of simple non-inflammatory softening, there was a large extravasation into one side of the brain, followed by sudden coma and hemiplegia. In the fourth and fifth cases there was sudden loss of consciousness, with convulsions, but no paralysis or contraction, and on dissection capillary apoplexy, with central softening, was found. In the sixth case, with extensive softening without effusion of blood, there was no disturbance of intellect, no contraction, no paralysis. Dr. Bennett considers that the softening arose from mechanical destruction of the tissue in the first three cases, and from post-mortem action in the three last.

Of the four cases in which both kinds of softening existed, in the first there was hemiplegia of the left side only. Softening was found in both corpora striata, but exudation corpuscles only in the right, the side opposite to the paralysis. In the second case there was impaired intelligence, loss of speech, disorganization of the eye, and convulsions before death; there was no paralysis. Abscesses surrounded by inflammatory softening were found in the external portion of the left anterior and middle cerebral lobes, explaining the symptoms present; but there was also non-inflammatory softening of the central parts of the brain producing no symptoms whatever. In the third case there was paralysis of both arms, contraction of the right, and tetanic spasms of the muscles of the mouth and neck. Inflammatory softening existed in the pons varolii, extending more to the left side; non-inflammatory softening of the right corpus striatum. In the fourth case there was headache, prominence of the eyeball, and coma, but no paralysis. A fungoid tumor was found at the base of the orbit, and an abscess in the anterior lobe of the brain, surrounded by inflammatory softening. There was also central yellow softening of the left hemisphere producing no symptoms.

Dr. Bennett considers that much of the obscurity which attaches to inflammation of the nervous centres arises from the fact that pathologists have hitherto confounded softening dependent on inflammation, with softening occasioned by post-mortem changes or mechanical violence, and, consequently, have been astonished to discover extensive softening after death, no corresponding symptoms having existed during life; on the other hand, where well-marked symptoms have been present, nothing has been discovered after death, though inflammation actually existed, capable of demonstration by the microscope alone, which shows the presence of exudation corpuscles.

Dr. Bennett next proceeds to make some remarks on the colour of softenings; the general relation between the symptoms and the seat of the lesion; on contraction of the limbs as a symptom of inflammatory softening; on the curability of softening; and on the connexion between softening and hæmorrhage.

1. *Colour of Cerebral and Spinal Softenings.*—Softening may be tinged red, yellow, white, or grey, without, however, any essential difference as to structure. As a general rule, it would appear that the red may be considered as acute, the yellow subacute, and the white or grey chronic softening; but this is by no means invariable.

The red and yellow colour is evidently connected with the presence of blood or its colouring matter. Sometimes the yellow softening is contained within the reddened portion, and somewhat resembles a purulent collection. At others, it surrounds the extravasation of blood, as is most common. Some have supposed that purulent infiltration is the cause of yellow softening, but such does not agree with the observations of Dr. Bennett, who has never met with a pus corpuscle in softened brain or spinal marrow.

In the fawn-coloured softenings, which are frequently observed independent of extravasated blood, the author has always found exudation corpuscles, and thinks it very probable that the fawn tint is attributable to the presence of these bodies, which are usually of a brownish or blackish colour.

White softenings are generally non-inflammatory; in some cases, however, they contain numerous exudation corpuscles, which are then colourless.

2. *General Relation between the Symptoms and the Seat of the Inflammatory Softening.*—In ten cases examined by Dr. Bennett, lesions of the central parts of the brain on one side were discovered; the symptoms during life consisted of contraction or paralysis of the extremities on the side opposite to the disease. In six cases there were lesions of the central parts on both sides; symptoms during life, contraction or paralysis on both sides of the body. In four cases, lesions of the peripheral parts only existed; this group was characterized by the absence of paralysis or contraction of the extremities, and by either delirium or coma. This analysis, it will be seen, is favourable to the hypothesis which ascribes motion and sensation to the central parts, intelligence to the surface.

3. *Contraction of the Limbs in Inflammatory Softening, and in Hæmorrhage.*—Some dispute has taken place as to the presence of more or less contraction or rigidity of the limbs in inflammatory softening, some authors contending that it is a highly characteristic symptom, others asserting that its presence, when it does occur, is altogether incidental. Dr. Bennett is of opinion that, in idiopathic inflammatory softening of the brain, contraction in one or more limbs is a common symptom. In *simple* hæmorrhage into the brain, on the other hand, without the existence of any inflammation, contraction seldom, if ever, occurs.

4. *The Curability of Cerebral and Spinal Softenings.*—Though numerous observations have fully demonstrated the possibility of this occurrence, Dr. Bennett considers that the anatomical marks or appearances, by means of which pathologists have endeavoured to demonstrate the fact, are very fallacious. "The slight indurations occasionally met with in the nervous substance are spoken of by some authors as *cicatrices*—a term, we think, wholly inapplicable to them. Durand-Fardel alludes to the softening resembling chalky milk as a proof of the passage of the lesion into a state of cure, and Dr. Sims speaks of the fawn-coloured cavities as evincing the same fact." In one case of hemiplegia of long standing, in which the chalky milk softening was found, the granules of the exudation corpuscles were seen to be large, equal in size, and very transparent, in fact presenting a very unusual appearance; it is not improbable, therefore, that the granules were undergoing absorption; and consequently the opinion of Durand-Fardel may be correct. On the other hand, the appearances described by Dr. Sims were met with in one case, but here, on the application of the microscope, numerous exudation corpuscles and granules were met with, precisely similar to those seen in parts undoubtedly affected with acute inflammation. Intense rigidity of the opposite side of the body also existed, without any other lesion than this which could at all account for it. Dr. Bennett's opinion therefore is, that the fawn-coloured spots described by Dr. Sims are no evidence of the cure of inflammatory softening.

5. *The Connexion between Softening and Hæmorrhage.*—It has long been a question, does the hæmorrhage precede and cause the softening, or does the softening ever precede and induce the hæmorrhage? In three cases related, hæmorrhage took place suddenly, producing hemiplegia, and death within a few hours; surrounding the coagulum the cerebral structure was softened, but apparently only mechanically, and no exudation corpuscles were found. In two other cases, with the same symptoms, death took place respectively in seven days and five weeks. Here the surrounding cerebral structure was softened, and numerous exudation corpuscles were discovered. Thus it would appear necessary for a certain time to elapse before the hæmorrhage can produce the inflammation. On examining the mode of accession also, there is no reason for supposing that softening preceded the hæmorrhage. In seven out of eight cases the attack was sudden, and in the eighth, where headache had existed for some time, this was evidently congestive, and the softening contained no exudation corpuscles or granules.

With regard to the character of the softening that frequently surrounds apoplectic clots or sanguineous infiltration, Durand-Fardel asserts, that if the softening extend beyond the limits of the infiltration, if redness surround it, and if enough blood be not infiltrated to have mechanically produced a diminution of consistence, then the affection is inflammatory. If, on the other hand, the softening is slight, its extent limited, if there be an adhesion of the meninges, and if the surrounding parts appear healthy, then the affection is simply sanguineous infiltration. With these distinctions, however, our author is not disposed to agree; two cases are brought forward, in one of which the appearances first mentioned were presented, whilst the second resembled what is described as simple infiltration. In neither case could any corpuscles or granules be discovered.

At this point Dr. Bennett pauses, promising however to renew the inquiry at some future period. The subject is one of the highest interest, and the observations of the author are deserving of the utmost attention.

---

### FACTS AND OBSERVATIONS RELATIVE TO THE INFLUENCE OF MANUFACTURES UPON HEALTH AND LIFE. By *Daniel Noble,* Esq.

THIS little pamphlet is in point of fact devoted entirely to the consideration of the sanatory influence of the cotton-manufacture. It is well known that, some years ago, a Committee of the House of Commons was appointed to enquire into what is called the "factory system." The medical evidence on that occasion was decidedly unfavourable; the general impression being that factory employment tended to produce diseases of the character and class ordinarily comprehended under the term scrofula; as impaired digestion, nervous debility, glandular disease, stunted growth, pulmonary consumption, &c. But to this evidence Mr. Noble objects that it was given principally by metropolitan practitioners who were necessarily ignorant of the actual working of the system, and who only speak of the effects of factory labour as it was represented to them; such representation being highly exaggerated with respect to the severity and hardship endured.

In 1835, a work, the Philosophy of Manufactures, was brought out by Dr. Ure, who took a very different view to that adopted by the Factory Commission, and one much more pleasing to the great cotton manufacturers. Unfortunately, however, it proved too much; not only was the ventilation of the mills highly superior—the temperature genial—the space for the workmen ample; not only was the factory system *not* prejudicial to health, and *not* tending to the development of scrofulous disease, but, on the contrary, it was actually preservative of the former, and, to some extent, curative of the latter. Nay more, according to Dr. Ure, at the time when the cholera was raging in England, the only class of persons that enjoyed immunity from the attack consisted of those employed in the cotton factories. Agricultural pursuits, though usually considered more healthy than employment in towns, ranked only secondarily in a sanatory point of view. In fact, after perusing the work, no one could doubt that the favourite residence of the Goddess of Health is a cotton-mill.

In 1840 was published the report of M. Villermé, who had been commissioned by the Academy of Moral and Political Sciences to enquire into the condition of the working classes. The report was, on the whole, favourable as regards the manufacture of cotton. Only one class in this branch of manufactures is reported to be unfavourably situated in a sanatory point of view, viz. the *batters of cotton*, whose occupation necessitates the inhalation of much dust and flue. These men complain of dryness in the mouth and throat, and are seized sooner or later with a cough gradually increasing in severity. This cough is the first symptom of a slow and formidable disease of the chest, which is always relieved on abandoning the work, and altogether cured at its commencement, if the employment be not resumed. The disease in its progress assumes all the characteristics of consumption.

Mr. Noble takes a middle course between the opinions of Dr. Ure on the one hand, and of the Factory Commission on the other. He cannot deny that the health of those engaged in factory-labour is more or less impaired, but he conceives that the great mass of disease which prevails in the manufacturing districts comes rather from the *great-town* than from the *factory* system. That is, although the position of the factory-operative is unfavourable to health and longevity, yet it differs but little, if at all, in this respect, from that of other classes of work-people who are exposed to the same injurious influences, such as the want of exercise, the crowding and imperfect ventilation of their residences, &c., excluding the effects, whatever they may be, which flow especially from the factory system. The Registration-returns demonstrate that, in this country, a densely-populated district is less favourable to life than one but thinly inhabited. Hence Mr. Noble contends, that the high mortality of manufacturing Manchester, as compared to agricultural Rutlandshire, (the average age of death among mechanics, labourers, &c. being in Manchester 17, in Rutlandshire 38), is owing not to the existence of the manufactures, but to the circumstance of the people being densely congregated. And, in support of this argument, he adduces the return of the mortality in Bethnal Green, a compact and thickly-populated part of the metropolis, but in which no factories exist; the average age of death of mechanics, labourers, &c. here, is 16! In Liverpool, the average age of death of the same class of persons is stated to be 15!

In the last chapter Mr. Noble considers in detail the specific ills which are said to result from the atmosphere of cotton-mills.

1st. *Acceleration of Puberty in the Female.*—This charge was probably founded on the idea, that the period at which maturity occurs in the female depends on the temperature of the place of habitation. This idea would however appear, from the researches of Mr. Roberton*, to be incorrect. Mr. Noble, from ample experience among factories, entertains the fullest conviction that this charge, at any rate, is unfounded.

2. *Bony Distortion.*—From a paper read at one of the meetings of the British Association it appeared that the cases of distortion in 640 factory children were 8, or 1¼ per cent. This average, it will be seen, is by no means high.

3. *Pulmonary Consumption.*—According to the Census of 1831, there were ascertained to be resident in Manchester and Salford 49,392 families; the number of deaths registered in 1839, amounted to 9,223, of which 1,454 are recorded as having been from consumption. This is at the rate of about 3 deaths annually from consumption to every 100 families, and of 15¾ per cent. of the deaths from all causes. From this it appears undoubtedly that consumption is exceedingly prevalent in these districts, for if we take Essex, an agricultural county, we find the cases of consumption to be not quite 2 annually, for every 100 families. But if we look at the proportion of deaths from consumption to those from other causes, we find it to be in Essex, 19 per cent, in the factory district only 16 per cent. In fact, with the exception of the Metropolis, Manchester has fewer deaths from consumption in proportion to the total number of deaths than most of the large towns, or even of the agricultural districts.

Mr. Noble has attempted to distinguish among the deaths from consumption that occur in Manchester, those that take place among the factory population; but we cannot say that we attach the slightest importance to his calculations, as they are entirely founded upon suppositions, and are, to say the least, much more likely to be wrong than right.

---

OBSERVATIONS ON PNEUMONIA AND ITS CONSEQUENCES. Read before the Physical Society of Guy's Hospital. By *Thomas Addison,* M.D. (Condensed from the Guy's Hospital Reports.)

THE object of this paper is to direct the attention of practitioners to a few points connected with the pathology, diagnosis, varieties and effects of Pneumonia. Dr. A. first considers that form of the disease which has been termed *Simple Pneumonia,* expressing some doubts, however, whether what has been so called is really the simplest form of the disease. Simple pneumonia is said to be characterized by certain symptoms, as fever, dyspnœa, pain of chest, and peculiar expectoration. The existence of cases, however, has been admitted in which these symptoms have been either entirely absent, or very ill-defined; in such cases, the disease has been termed *latent pneumonia,* and when marked, *typhoid pneumonia.* The general impression is that, whenever pneumonia occurs in young and robust constitutions, these symptoms must be present. This opinion is at variance with Dr. Addison's experience; he considers, in fact, that the simple pneumonia of Laennec and others is by no means the simplest form of the disease, but a complication, a *broncho-pneumonia;* the truly simple pneumonia occasionally occurring in young persons and in good constitutions, unattended by any of the above-named symptoms. With respect to the *seat* of pneumonia considerable diversity of opinion has existed and even still exists; some asserting it to be the so-called parenchyma of the lung; while others, and among them Dr. Addison, will have it that pneumonia has its original and essential seat in the air-cells of

* Med Chir. Review, Oct. 1843.

the lungs, and that the ordinary pneumonic deposits are poured into these cells, and not into an interstitial tissue or parenchyma, of which structure he professes himself unable to find a single trace. With respect to the nature of the elementary tissues composing the air-cells* of the lungs, and the exact construction and arrangement of these cells, he has been unable to arrive at any positive conclusion—he is fully persuaded, however, that *pathologically* they present none of the attributes of a mucous membrane; and if the pathological change induced in any tissue constitutes a legitimate foundation for an opinion respecting the physiological character of that tissue, one is almost irresistably led to the conclusion that the air-cells of the lungs are a mere modification of areolar tissue, or of a serous membrane. The effect which immediately succeeds the earliest stage of pneumonic inflammation wherein there is supposed to be a preternatural dryness of the air-cells from an arrest of their natural secretions—an effect, too, which is usually the first recognised by physical signs, being the so-called state of engorgement, seems most strongly to countenance the belief in the serous character of the air-cells. This effect consists in the effusion of serum into the cavities of the cells themselves. In this stage, together with a highly vascular condition of the cells, the cells are found to be denser to the feel than natural; subsequently the turgid parietes of the cells lose their natural cohesion, swell, and, encroaching on the cavities of the cells, cause the absorption of the effused serum, and, by thus filling up these cavities, occasion, first a brittleness, and afterwards the consolidation which constitutes red hepatization—a consolidation depending not on an effusion of solid albuminous matter into the cells, but on the great vascularity, softness, and tumefaction of the cells themselves.

The subsequent changes produced by inflammation consist not only in a loss of cohesion and more or less tumefaction of the inflamed tissue, but in a remarkable disposition in that tissue to return to a state more or less resembling that which forms the original base of all tissues; viz. an albuminous material. This reconversion of a tissue into albumen, Dr. A. calls *albuminization*—hence, as the inflammation proceeds, the parietes of the cells become more opaque; the minute vessels are no longer visible; the parietes of the cells become thickened and softened, so that the cells now admit of an albuminous matter being poured into their cavities. This constitutes the grey hepatization of authors. When the inflammation occurs in good constitutions, this albuminous matter may be more or less solid; when, however, it attacks a bad constitution and assumes an atonic form, the albuminous effusion is more fluid, and may be readily forced out by gentle pressure, or by pricking the containing cells. These changes may, in any state of constitution, take place in a considerable extent of pulmonary tissue, obliterating or concealing the lobes of common cellular membrane, separating one lobule from another. In other cases, however, and especially in certain atonic forms of pneumonia, these changes are confined to individual lobules, more or less remote from each other, the common cell-membrane forms a distinct boundary to the inflammation—this is called *lobular pneumonia.* It has been long remarked that acute pneumonia very rarely terminates in abscess; and in atonic inflammation attacking bad constitutions, though abscesses are not quite so rare, they are exceedingly small and regularly scattered through the inflamed part. Gangrene, more especially in its diffuse form, is acknowledged to be still more rare; when it does occur in this form, it appears to be closely allied to sloughing; there is another form of gangrene, called circumscribed gangrene; this appears to be the result of pulmonary apoplexy. *Lobular pueumonia,* as abovementioned, is for the most part observed in cachectic habits of body, and especially towards the termination of various chronic diseases, after surgical

* For the latest information on this subject see a Paper by Mr. (not Doctor) Addison, in the Philosophical Transactions for 1842, Part 2.—*Rev.*

operations, and in phlebitis. The abscess in such cases is sometimes of considerable size—very complete and very rapidly formed, attributable, no doubt, to the rapid exhaustion of the vital properties of the inflamed tissue.

There is another form of consolidation of the lung, called by Laennec *carnification*, which is generally regarded as the result of pneumonic inflammation, modified by pleuritic effusion. Dr. Addison cannot subscribe to this opinion; he feels satisfied that it results from pleuritic effusion having compressed the lung, forced out the air, and thus brought the sides of the cells into close contact; he does not, however, deny that it may be occasionally blended with pneumonic inflammation and its consequences.

Dr. Addison now comes to consider the remote consequences of pneumonia. The *permanent* effects produced in a lung by pneumonia depend chiefly on the state of the patient's constitution, and the character of the inflammation, and consequently on the nature of the albuminous deposits, as also on the degree of organization of which such deposit is susceptible. Of these effects he lays down three varieties: 1. *The uniform albuminous induration.* 2. *The granular induration.* 3. *The grey induration.*

When acute pneumonia occurs in good constitutions, the softened tissues become so assimilated to the permanent albuminous deposit, that the whole is converted into a uniform homogeneous material, in which there is not the slightest trace of either the aerial cellular structure, or of the common interlobular cellular membrane. This may be either diffused over several lobules; or, what is more rare, it may be confined to one or a very few only. This, the least frequent of the permanent pneumonic indurations, he calls *uniform albuminous induration.*

When, again, the inflammation pours out a less organizable albumen, as happens in strumous habits, the change produced is very different. Here, in general, the interlobular cellular tissue remains perfectly distinct; a solid, pale, or yellowish and friable albuminous matter occupies the lobules; and this without having assimilated the parietes of the cells. The cellular arrangement is still perceptible; whilst on cutting the lobule we find the friable albuminous matter presenting the granular aspect produced by the filling up of still separate cells. This constitutes the *granular induration.* It resembles in its appearance ordinary tuberculous matter; whence it is sometimes called *inflammatory tubercle.*

The *grey variety* of pneumonic induration is next considered: this morbid change differs from the uniform albuminous induration in the albuminization of the tissues being much less complete—from the granular induration it differs in the albuminous deposit being of a more plastic or organizable kind; in short, the pneumonic inflammation may be said, in this case, to have terminated in adhesion: some assert that this grey or black induration of the lung results from chronic pneumonia. As the morbid changes now described have been indiscriminately considered as forms of tubercular infiltration, it may be asked, what proof have we of their inflammatory origin. To this Dr. Addison replies, 1st. That we occasionally have proofs of a previous attack of acute inflammation within the chest. 2d. They are uniformly accompanied by evidences of former inflammation; viz. old deposit on the pleura immediately above the affected portion of the lung; adhesion between the pleuræ in that situation, &c. 3rd. They are generally found in the middle and inferior lobes of the lung: and, lastly, the total absence, in many instances, of a vestige of tubercle in other parts of the lung. Dr. Addison now adverts to the question regarding the relation which exists between pneumonic deposits and tubercle—whether tubercle be uniformly and necessarily the result of a process of inflammation; or if not, whether it is so in any, and in what instances. Without pretending to remove the difficulties in which this subject is still involved, he proceeds to state the conclusions to which he has been led by his inquiries and experiments.

He first notices the general resemblance observed to exist between the effects of tubercular disease occurring in serous membranes, and those met with in the lungs; the several forms, changes, and varieties presented by the one being almost equally observable in the other. The earliest and simplest form of tubercular disease in a serous membrane consists of a minute, roundish, semi-transparent projection, hard to the touch and very tenacious; in like manner, the earliest and simplest form presented by tubercles in the lungs is that of minute grey semi-transparent hard bodies, inseparably attached to the parietes of the air-cells of the lungs, in the same manner as the small tubercles are incorporated with the peritoneum. Neither in the case of the peritoneum, nor in that of the lung do we at this early stage discover the slightest trace of inflammatory action. Yet we know from abundant facts that the peritoneum, when once tuberculated, is very liable to inflammation; the effects of which are opacity, thickening, and contraction of the membrane; in the lungs we find a state very similar to this. In certain lobules, or in a single lobule, we find a cluster of tubercles, presenting now a more dull or opaque appearance; this change is followed by a decided attempt at contraction or puckering of the affected lobule from a cause probably similar to that in the peritoneum. (For the very minute description of the morbid changes which take place in the tubercular deposits in the lung and in the serous membranes, we beg to refer the reader to the original paper.)

Independently of the arrest of secretion said to attend the onset of inflammation in every structure, the ordinary morbid changes immediately produced by pneumonia are, 1st. An effusion into the air-cells of serum, more or less highly charged with albumen in solution; 2. A molecular change in the inflamed tissue, characterized by more or less opacity and loss of cohesion. 3. A deposition of albumen into the cells, either solid and organizable, or fluid and puriform. 4. Total albuminization of the tissues, either in the form of a material susceptible of organization, or of a material unsusceptible of organization, and thence forming an abscess. On the other hand, the salutary or reparatory changes are, 1. Absorption of the effused serum. 2. Such a change in the molecular condition of the tissue as restores to it its natural cohesion, and its transparency. 3. Organization of the effused albuminous matter, with consequent contraction and induration of the tissues into which it is effused, or absorption of the albuminous effusion, if puriform. 4. Organization of the albuminized tissue, when susceptible of that change; or the formation of a cyst to circumscribe the abscess, when the conversion is of the puriform kind. The immediate morbid changes produced by ordinary pneumonia and by phthisical disease are the same, with the exception of the albumen, whether effused, or resulting from albuminization of the tissues, being much more susceptible of organization, and consequently more likely to become permanent in the former than in the latter; whereas, the reparatory changes are less complete, and less permanent in phthisis than in pneumonia. Hence, according to Dr. Addison, the uniform albuminous induration can scarcely ever be said to have occurred in strongly-marked phthisical disease, whilst the granular induration may be said to form a connecting link between phthisical disease and ordinary pneumonia. The grey induration in phthisical subjects is much less firm, much less permanent, and generally soon softens down into a vomica or abscess; so that, as we speak of scrofulous peritonitis, we may, in designating tubercular phthisis, call it scrofulous pneumonia. A plausible objection has been made to the inflammatory origin of tubercular disease, viz. that it most frequently takes place in the apices of the lungs, whereas the reverse holds good in reference to pneumonia. As a set off against this objection, Dr. A. says that, in his own experience, on examining the lungs of individuals who have died of disease not affecting the chest at all, he has found indications of partial inflammation much more frequently towards the apices, than in the lower portions of the lungs. Partial pleuritic adhesion and pneumonic changes, in his experience, are more frequently met in the former than in the latter situation. This, he conceives, may go some way to account for the earlier development of tubercular disease in the former than in the latter situation. The several morbid structures or deposits that have been alluded to in this paper maintain their integrity by a very slender power: the degree of vital influence which holds their molecules in a solid state, being impaired or destroyed by very slight disturbing causes; the most common of these causes being inflammation set up in the surrounding parts, and a cachectic habit of body, but more especially these two causes occurring in the same individual. Under these circumstances, those structures and deposits soften down, and are gradually converted into an albuminous or puriform fluid, and thus give rise to the symptoms of phthisis.

(As we are promised a continuation of this very interesting paper, we shall discontinue our analysis for the present to resume it when the paper shall have been completed.)

---

Minor Surgery; or Hints on the Every-day Duties of the Surgeon. By *Henry H. Smith*, M.D. &c. Illustrated by Engravings. Philadelphia; Ed. Barrington and Geo. D. Haswell. 1843.

Minor Surgery is in practice Major Surgery, reversing the scholastic axiom that omne majus in se continet minus. For every surgeon and every practitioner has a great deal of the minus and very little, perhaps none, of the majus. A man may prepare himself to tie aneurysms, amputate limbs, trephine crania, in fact perform human vivisections on a large scale, and never get a patient on whom to flesh his knife. The very same man may not think it worth his while to learn how to bandage, dress, and treat those common cases which form the great bulk of the highest practice, and the whole of the practice of most people. What is the consequence? Daily ignorance, mortification, and loss, or, at all events, not gain, of reputation.

We wish our students could be induced to think of these matters. They are caught by glare, and run after the great operations. The unobtrusive treatment of disease has little charm for them—the out-patients of Hospitals, and the patients of Dispensaries are neglected—they *would* be Clines and Coopers, and they do *not* make themselves good practitioners.

Such books as those before us are calculated to meet this crying evil. Its author informs us:—

"The shortness of the period usually allotted to a course of lectures on Surgery, and the rapidity with which the lecturer is obliged to pass over the methods of Dressing and the Minor Surgical Operations, has left a deficit in the amount of knowledge required for daily practice, which every one commencing has more or less severely felt. With a view of filling up this, as well as in compliance with the repeated requests of several members of his class, the author has been induced to undertake the present work, not in the expectation of being able to offer anything new or original on a subject which has so long engaged more or less of the attention of every one, but with the hope that he might afford a concise and methodical system of Minor Surgery."

And a capital little book it is. Surgical authors have been freely laid under contribution—wood-cuts in profusion (not *quite* equal to Baggs's) illustrate the text, and stand for it—bandages are drawn with reckless prodigality—fingers and thumbs occupy every conceivable position—there is a history of *cravats* which would puzzle Beau Brummell—and diagrams which even shew us how to cut a strip of sticking plaister, and put a leech in a glass.

And all this is as it should be. Little matters are the sum of professional, as of private life, and its comfort and happiness are mixed up with their management, be it right or wrong. Minor Surgery, then, we repeat, is really Major Surgery, and anything which teaches it is worth having. So we cordially recommend this little book of Dr. Smith's.

